# Patient-reported outcome measures in functional dyspepsia: a systematic review and COSMIN analysis

**DOI:** 10.1186/s12876-023-02935-9

**Published:** 2023-09-19

**Authors:** Xinai Wang, Yan Fei, Wenjing Li, Hao Liu, Hongling Xiao, Yaxuan Wu, Chenqi Wang

**Affiliations:** 1 The School of Graduate, Tianjin University of Traditional Chinese Medicine, Tianjin, China; 2 Shanghai Tenth People’s Hospital Chongming Branch, Shanghai, China; 3 The School of Nursing, Tianjin University of Traditional Chinese Medicine, Tianjin, China

**Keywords:** Functional dyspepsia, Dyspepsia, Patient-reported outcome measure, Measurement properties

## Abstract

**Background:**

Functional dyspepsia (FD) as a type of disorders of brain-gut interaction (DBGI), patient self-reporting of its symptoms becomes an important component of clinical outcome assessment. We performed a systematic review using Consensus Based Standards for the Selection of Health Measurement Instruments (COSMIN) guidelines to identify the best available patient-reported outcome measure (PROM) of FD.

**Methods:**

The study was conducted according to the Preferred Reporting Items for Systematic Reviews and Meta-Analyses (PRISMA). We searched four databases with no date limit, looking for previously confirmed PROMs for evaluating FD symptoms. An overall rating was then assigned based upon COSMIN guidelines, and the Grading of Recommendations Assessment, Development, and Evaluation (GRADE) approach was used to assess the level of evidence for psychometric properties of included PROMs.

**Results:**

Thirty articles covering outcome indicators of 24 patient reports were included. The Leuven Postprandial Distress Scale (LPDS) showed adequate content validity and moderate quality evidence of adequate internal consistency to generate an A recommendation.

**Conclusion:**

LPDS is currently the most recommended PROM for patient self-reported FD symptoms. However, it fails to assess two important areas of cross-cultural validity/ measurement invariance and measurement error. Future research can be continuously improved on this basis.

**Supplementary Information:**

The online version contains supplementary material available at 10.1186/s12876-023-02935-9

## Introduction

Functional gastrointestinal disease as a chronic disease is a very common clinical condition encountered in the clinical practice of gastroenterology, and the Rome IV redefines functional gastrointestinal disease as disorders of gut-brain interaction [1]. Functional dyspepsia (FD) is one of its most common types, divided into postprandial distress syndrome (PDS) and epigastric pain syndrome (EPS). Early satiation and postprandial fullness are the main symptoms of PDS, while epigastric pain and epigastric burning in the upper abdomen are the main symptoms of EPS. In addition, upper abdominal bloating, postprandial nausea and excessive belching are also considered important additional symptoms of FD [2, 3]. The prevalence of functional dyspepsia is 10–30% worldwide and varies between regions, with a higher average prevalence in Asia than in Europe and the Americas [4–6]. And under the influence of the COVID-19, the incidence and recurrence rates of the disease have increased significantly, consuming a large amount of medical resources and placing a heavy burden on the health care system [7]. The high incidence and persistence of FD seriously affects the quality of life of patients. However, the pathogenesis of FD has not been fully elucidated so far, and it is generally believed to be associated with multiple pathophysiological mechanisms, with gastrointestinal motility disorders, visceral hypersensitivity, immune dysfunction, altered gastrointestinal microbiota, abnormal gut-brain interactions, psychosocial factors, and genetic susceptibility as existing etiologies [8]. Because of its complex pathogenesis, the clinical use of pro-gastrointestinal drugs, antacids and antidepressants and other drug therapy has a positive effect. In addition, non-pharmacological treatments such as acupuncture and hypnosis may be effective, but the corresponding research is scarce [9–12].

Efficacy assessment as an important indicator to evaluate the effectiveness of treatment regimens has been an aspect of general interest in various research areas. However, up to now, there is no "gold standard" for assessing the effectiveness of FD treatment protocols, and the most appropriate assessment scale remains to be determined. FD has been suggested as a group of symptoms originating in the gastric and duodenal regions, without underlying organic, systemic or metabolic disease that might explain the symptoms [13]. This is why patients' self-reporting of their symptoms becomes an important element of efficacy assessment and is more authentic and credible. The Food and Drug Administration (FDA) Guidelines similarly recommend the use of well-defined patient-reported outcome measures (PROMs) to assess treatment outcomes [14]. A recent systematic evaluation summarized the prom of symptom assessment in patients with FD from 1970 to 2017, but has not assessed the risk of bias and level of evidence for psychometric properties [15]. According to COSMIN guidelines [16] and GRADE approach [17], this study attempts to comprehensively analyze the PROMs included in the study, and formulate its recommendation level, so as to select the best PROMs for evaluating FD symptoms and provide reference for clinicians to choose an appropriate curative effect evaluation scale.

## Methods

### Data sources and search strategy

**Fig. 1:**
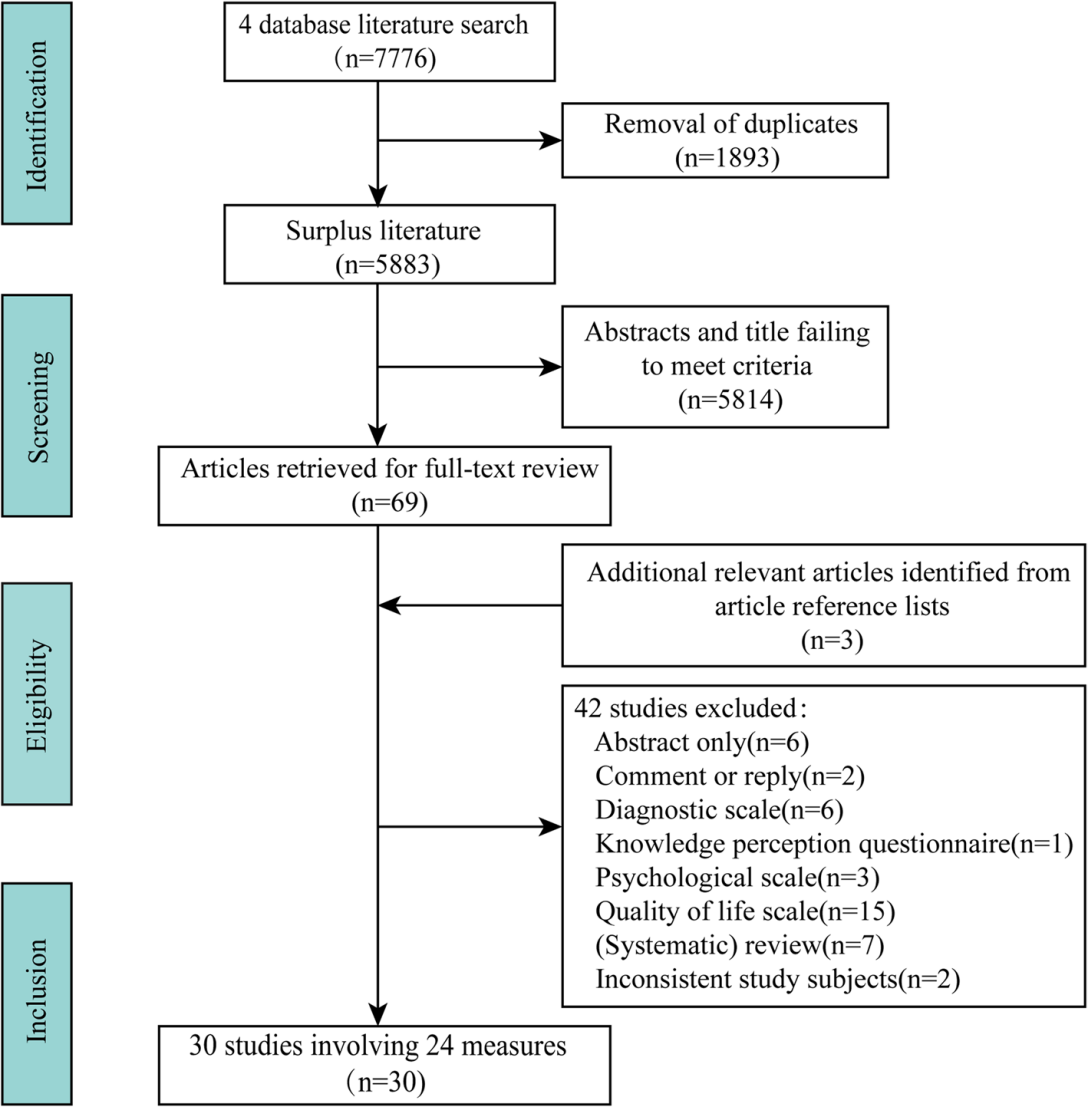
Flow diagram of the search and selection process

One researcher conducted a literature search in PubMed, Cochrane, Embase, and Web of science databases on October 7, 2022, with no date restrictions and using English as a filter. The search formula for each database is shown in Supplementary Material 1. In addition, the researchers checked the reference list of the literature read in full for additional relevant citations (Fig. 1).

### Inclusion and exclusion criteria

Studies were original literature from PROMs or psychometric literature, applicable to adult patients with a confirmed FD and published publicly in academic journals in English. Studies were excluded if they were reviews, commentaries or responses to the original study, or abstracts without the full text (presented at a conference). In addition, studies that were not symptom assessments (e.g., scales used to diagnose FD) or that assessed only quality of life, perceived knowledge, or psychology were excluded (Fig. 1). Titles and abstracts found in the literature search were judged independently by two researchers, and for the remaining full-text articles were also independently searched and judged eligible by two reviewers. Both researchers agreed on the inclusion of these articles, and if any inconsistencies arose, agreement was reached by consulting a third researcher. Full-text articles were screened if at least one researcher considered a study relevant based on the abstract, or if there was doubt.

### Data analysis

After removing duplicates from four different databases using Endnote software, three researchers extracted data from the included studies using a standardized data collection tool. Two researchers independently evaluated the psychometric properties and methodological quality of the included PROMs using the COSMIN systematic review guidelines [16] and cross-checked the results. The GRADE approach was then used to synthesize the level of evidence for the inclusion of each PROMs and to form a final recommendation for the scale. In case of disagreement, it was resolved through consultation and agreement with a third researcher.

1. COSMIN Risk of Bias checklist [18]: COSMIN Bias Risk Checklist is a part of COSMIN Guidelines. The methodological quality of the included scales was assessed using the COSMIN Risk of Bias Inventory in five dimensions: very good (V), adequate (A), doubtful (D), insufficient (I), or not assessed (NA). If the risk of bias differs across entries in a measurement characteristic, the overall risk of bias rating (related to the method used) is assessed based on the "lowest score count" principle.
2. COSMIN quality criteria [19]: COSMIN quality criteria is another part of COSMIN guidelines. The psychometric characteristics of the included scales were assessed using the COSMIN quality criteria, and the included PROMs were rated as adequate ( +), inadequate (-), inconsistent ( ±), or uncertain (?) based on the results reported in the individual studies. If only one study assessed the psychometric properties of a scale, it was used as an overall quality assessment; if multiple studies jointly assessed a particular psychometric characteristic of a scale, their individual scores were aggregated to provide an overall quality assessment of the instrument.
3. GRADE approach [17]: the COSMIN modified GRADE was used to assess the level of evidence of the included scales into 4 dimensions: high (High), moderate (Moderate), low (Low) and very low (Very low).COSMIN assumes that each measured characteristic is of high quality and then downgrades them according to the following 4 components: (i) risk of bias (if there is a serious, very serious, or extremely serious risk of bias, then downgrade by one, two, or three levels, respectively); (ii) inconsistency (if the inconsistency is serious or very serious, then downgrade by one or two levels, respectively); (iii) imprecision (if the sample size is between 50 and 100, then downgrade by one level; if the sample size is < 50, then downgrade by two levels; not applicable to content validity, structural validity, and cross-cultural validity); (iv) indirectness (if it is serious of or very serious indirectness, then the grade was lowered by one or two levels, respectively). Evidence recommendations are divided into three categories: A, B, and C. Level A: has "sufficient ( +)" content validity (for any level of evidence) and "sufficient ( +)" internal consistency (for at least low quality levels of evidence); Level C: has high quality evidence of "inadequate (-)" measurement properties; Level B: Level B if it does not belong to Levels A and C.

## Results

Seven thousand seven hundred seventy-six literatures were retrieved from four databases, and 5883 remained after excluding 1893 duplicate literatures; then, each paper’s title and abstract was read one by one, and finally, 69 articles met the inclusion criteria. After reading the full text and further screening the literature, 30 studies including 24 FD symptom assessment scales were finally included [20–49]. The details of the selection process can be seen in Fig. 1.

### Summary of study data

The general information on the included PROMs is shown in Table 1. The included studies were divided into developmental, validation and intervention studies, the number of patients assessed ranged from 35–1633. Their publication countries were dispersed, with the most scales studied and published in the USA (DHSI, DSSI, SODA, GISSI and FDSD), followed by the UK (GDSS, CDQ, LDQ and SF-LDQ). The number of entries for each PROMs ranged from 5 to 40, with the NDI scale having the highest number of entries, which assessed both symptom and quality of life components; the longest recall period was 24 weeks (GDSS and LDQ), and the shortest recall period was the ESM-PROM which is a real-time assessment. Multiple translated versions are available for the following included PROMs: GSRS, DHSI and NDI (8 translations) and PAGI-SYM (7 translations). English translated version is the most common version in included PROMs (21 of 24), and 5 PROMs (GDSS, LDQ, GIS, FDSD, and ESM-PROM) reported the fill time, but the rest were not mentioned. 14 PROMs were scored using Likert scale (4–7 points), 2 PROMs used an 11-point numeric rating scale (NRS), and the remaining studies used Rasch model calibration scales, images, or smiley faces for outcome measures. More detailed information can be seen in Table 1.

**Table 1:** Details of the findings

Scale	Time	Country	Number of items	Recall period	Patients studies	Translated version	Fill in time	Outcome measurement
GSRS	1988–2008	Sweden	15	1 week or 4 weeks	204–853	English, Dutch, German, Hungarian, Italian, Polish, Spanish, Navajo	Undefined	Likert-4
GDSS	1996	United Kingdom	8	24 weeks	230	English, Spanish	4 min	Undefined
CDQ	1996	United Kingdom	15	2 weeks	287	English	Undefined	Undefined
LDQ	1998	United Kingdom	9	24 weeks	215	English, Chinese, Italian, Farsi	5 min	Likert-5
DHSI	1998–2001	United States	34	4 weeks	690–692	English, Malay, Norwegian, French, Dutch, Italian, German, Spanish	Undefined	Undefined
NDI	1999	Australia	Quality of life component: 25 Symptom checklist: 15	2 weeks	113	English, French, Dutch, Italian, German, Spanish, Chinese, Arabic	Undefined	Likert-5
DSSI	2000	United States	20	2 weeks	72	English	Undefined	Likert-5
SODA	2001	United States	17	1 week	98	English, Spanish	Undefined	Rasch model
SLDQ	2002	Spain	38	Undefined	63	Spanish	Undefined	Likert-6
HKDI	2002	Hong Kong, China	12	Undefined	130	Chinese	Undefined	Likert-5
PADYQ	2004	Brazil	11	4 weeks	62	English, Brazilian Portuguese	Undefined	Undefined
PAGI-SYM	2004	Germany	20	2 weeks	767–1577	English, French, German, Italian, Polish, Dutch, Spanish	Undefined	Likert-6
Severity Index of Bologna	2004	Italy	8	Undefined	148	Italian	Undefined	Likert-5
GIS	2005	Australia	10	Undefined	151	English	2–3 min	Likert-5
GOS	2006	Canada	10	2 days or 4 weeks	1633	English	Undefined	Likert-7
SF-LDQ	2007	United Kingdom	5	8 weeks	592	English, Indonesian, Urdu	Undefined	Likert-5
GISSI	2015	United States	39	2 weeks or 30 days	934	English	Undefined	Likert-5
LPDS	2014–2016	Belgium	8	1 day	91–229	English, Dutch, French	Undefined	smiley faces
FDSD	2018–2022	United States	8	1 day	102–512	English	1 min 30 s	NRS
SAGIS	2017	Australia	22	1 week	1120	English	Undefined	Likert-5
ESM-PROM	2019–2021	Netherlands	33	Real Time	35–45	English, Dutch	3-5 min	NRS
FGI-Checklist	2020	Hong Kong, China	20	1 week	641	English, Chinese	Undefined	Likert-4
a Scale for PDS	2021	China	Undefined	1 day	≥ 100	Chinese	Undefined	smiley faces + VAS
SEQ-DYSPEPSIA	2022	Korea	11	2 weeks	193	English, Korean	Undefined	Likert-5 + cartoon-style images

*GSRS* Gastrointestinal Symptom Rating Scale, *GDSS* Glasgow Dyspepsia Severity Index, *CDQ* The Clinical Dyspepsia Questionnaire, *LDQ* Leeds Dyspepsia Questionnaire, *DHSI* Digestive Health Status Instrument, *NDI* Nepean Dyspepsia Index, *DSSI* Dyspepsia Symptom Severity Index, *SODA* Severity of Dyspepsia Assessment, *SLDQ* Spanish Language Dyspepsia Questionnaire, *HKDI* Hong Kong Dyspepsia Index, *PADYQ* Porto Alegre Dyspeptic Symptoms Questionnaire, *PAGI-SYM* Patient Assessment of Upper Gastrointestinal Symptom Severity Index, *GIS* Gastrointestinal Symptom Score, *GOS* Global Overall Symptom Scale, *SF-LDQ* Short-form Leeds Dyspepsia Questionnaire, *GISSI* Gastrointestinal Symptom Severity Index, *LPDS* Leuven Postprandial Distress Scale, *FDSD* Functional Dyspepsia Symptom Diary, *SAGIS* Structured Assessment of Gastrointestinal Symptoms Scale, *ESM-PROM* Experience Sampling Method-Patient Reported Outcome Measure, a Scale for *PDS* a Scale for Post-prandial Distress Syndrome, *SEQ-DYSPEPSIA* Self-evaluation Questionnaire for Functional Dyspepsia

### Content validity assessment

Content validity includes relevance, comprehensiveness and comprehensibility. Whether PROMs assessed all symptoms of FD is one aspect of content comprehensiveness (Table 2). The included PROMs were evaluated based on FD symptoms as defined by the Rome IV. Seven PROMs (NDI, DSSI, LPDS, FDSD, ESM-PROM, FGI-Checklist and SEQ-DYSPEPSIA) scales can evaluate four core symptoms and three additional symptoms of FD, followed by GOS and GISSI, which can evaluate six discomfort symptoms. The remaining PROMs scales all assessed symptoms incompletely.

**Table 2:** Comprehensiveness of the content incorporated into the scale

Patient-reported outcome measures	Core concepts from Rome IV				Additional concepts from Rome IV			Total (*N* = 7)
	Epigastric pain	Epigastric burning	Postprandial fullness	Early satiation	Upper abdominal bloating	Postprandial nausea	Excessive belching	
GSRS	√				√	√	√	4
GDSS								0
CDQ								0
LDQ	√			√		√	√	4
DHSI			√	√	√	√		4
NDI	√	√	√	√	√	√	√	7
DSSI	√	√	√	√	√	√	√	7
SODA	√					√	√	3
SLDQ	√			√	√	√		4
HKDI	√	√			√	√	√	5
PADYQ	√			√	√	√		4
PAGI-SYM	√		√	√	√	√		5
Severity Index of Bologna	√				√			2
GIS	√			√	√	√		4
GOS	√		√	√	√	√	√	6
SF-LDQ						√		1
GISSI	√		√	√	√	√	√	6
LPDS	√	√	√	√	√	√	√	7
FDSD	√	√	√	√	√	√	√	7
SAGIS	√			√	√	√	√	5
ESM-PROM	√	√	√	√	√	√	√	7
FGI-Checklist	√	√	√	√	√	√	√	7
a Scale for PDS			√	√				2
SEQ-DYSPEPSIA	√	√	√	√	√	√	√	7

The overall evaluation of the content effectiveness is shown in Table 3. The overall content validity of these studies was unsatisfactory for reasons including, but not limited to, insufficient relevance or comprehensiveness of unreported content. Based on the COSMIN risk of bias assessment checklist, the methodological quality of most of these studies was found to be reported as grade D (doubtful) for content validity, with HKDI, Severity Index of Bologna and GOS rated as grade I (insufficient). The comprehensiveness of the content was compromised because most scales did not fully assess the core and additional symptoms of functional dyspepsia, and the comprehensiveness of the final content comprehensiveness was mostly ± (inconsistent). Only 7 PROMs (NDI, DSSI, LPDS, FDSD, ESM-PROM, FGI checklist, and SEQ-DYSPEPSIA) comprehensively assessed all symptoms of FD with a content validity in comprehensiveness of + (adequate). However, the correlation and comprehensibility of the DSSI, FGI-Checklist and SEQ-DYSPEPSIA were insufficient or have not been reported, so the final content validity score was ± (inconsistent). Most of the included studies had a low level of evidence for content validity.

**Table 3:** Summary of findings. (M: Methodology reported as ‘very good(V)’, ‘adequate(A),’ ‘doubtful(D)’, ‘inadequate(I)’, or ‘not assessed (NA)’. R: Ratings for overall quality reported as sufficient ( +), insufficient (-), inconsistent ( ±), or indeterminate (?). L: Level of evidence using GRADE reported as: High, Moderate, Low, or Very low.)

Patient-reported outcome measures	Content validity			Structural validity			Internal consistency			Cross-cultural validity/ measurement invariance			Reliability			Measurement error			Hypothesis testing			Responsiveness			Recommendation
	M	R	L	M	R	L	M	R	L	M	R	L	M	R	L	M	R	L	M	R	L	M	R	L	
GSRS	D	±	Low	I	?	Very low	V	?	Moderate	NA	?	NA	A	-	Low	NA	?	NA	V	+	Moderate	NA	?	NA	B
GDSS	D	±	Low	NA	?	NA	NA	?	NA	NA	?	NA	NA	?	NA	NA	?	NA	NA	?	NA	V	?	High	B
CDQ	D	±	Low	A	-	Moderate	V	+	High	NA	?	NA	A	-	Moderate	NA	?	NA	V	+	High	NA	?	NA	B
LDQ	D	±	Low	NA	?	NA	V	?	High	NA	?	NA	A	+	Moderate	NA	?	NA	NA	?	NA	V	?	High	B
DHSI	D	±	Low	A	?	Moderate	V	-	High	NA	?	NA	A	-	Moderate	NA	?	NA	V	+	High	V	+	High	C
NDI	D	+	Low	A	?	Moderate	NA	?	NA	NA	?	NA	NA	?	NA	NA	?	NA	NA	?	NA	NA	?	NA	B
DSSI	D	±	Low	I	?	Very low	V	+	Moderate	NA	?	NA	A	+	Low	NA	?	NA	NA	?	NA	NA	?	NA	B
SODA	D	±	Low	A	+	Moderate	V	+	Moderate	NA	?	NA	NA	?	NA	NA	?	NA	NA	?	NA	V	+	Moderate	B
SLDQ	D	±	Low	I	?	Very low	V	?	Moderate	NA	?	NA	A	+	Low	NA	?	NA	V	+	Moderate	V	+	Moderate	B
HKDI	I	±	Very low	I	?	Very low	V	?	High	NA	?	NA	A	+	Moderate	NA	?	NA	V	±	High	V	?	High	B
PADYQ	D	±	Low	D	?	Low	V	+	Moderate	NA	?	NA	A	+	Low	NA	?	NA	V	?	Moderate	V	?	Moderate	B
PAGI-SYM	D	±	Low	A	?	Moderate	V	+	High	NA	?	NA	A	-	Moderate	NA	?	NA	V	+	High	V	?	High	B
Severity Index of Bologna	I	±	Very low	NA	?	NA	NA	?	NA	NA	?	NA	A	-	Moderate	NA	?	NA	NA	?	NA	V	?	High	B
GIS	D	±	Low	D	?	Low	NA	?	NA	NA	?	NA	A	+	Moderate	NA	?	NA	NA	?	NA	V	?	High	B
GOS	I	±	Very low	I	?	Very low	NA	?	NA	NA	?	NA	A	-	Moderate	NA	?	NA	V	?	High	V	?	High	B
SF-LDQ	D	±	Low	NA	?	NA	V	?	High	NA	?	NA	D	?	Low	NA	?	NA	NA	?	NA	V	?	High	B
GISSI	D	±	Very low	V	+	Moderate	V	-	Moderate	NA	?	NA	A	+	Low	NA	?	NA	NA	?	NA	NA	?	NA	B
LPDS	D	+	Low	V	?	High	V	+	Moderate	NA	?	NA	I	?	Very low	NA	?	NA	V	+	Moderate	V	?	Moderate	A
FDSD	D	+	Low	NA	?	NA	V	?	High	NA	?	NA	NA	?	NA	NA	?	NA	V	?	High	NA	?	NA	B
SAGIS	D	±	Low	V	-	High	V	+	High	NA	?	NA	I	?	Very low	NA	?	NA	V	+	High	NA	?	NA	B
ESM-PROM	D	+	Low	NA	?	NA	V	?	Low	NA	?	NA	A	+	Very low	NA	?	NA	NA	?	NA	NA	?	NA	B
FGI-Checklist	D	±	Low	A	?	Moderate	V	-	High	NA	?	NA	NA	?	NA	NA	?	NA	V	?	High	V	+	High	C
a Scale for PDS	D	±	Low	NA	?	NA	NA	?	NA	NA	?	NA	NA	?	NA	NA	?	NA	NA	?	NA	NA	?	NA	B
SEQ-DYSPEPSIA	D	±	Low	I	?	Very low	V	?	High	NA	?	NA	A	+	Moderate	NA	?	NA	V	?	High	V	?	High	B

### Assessment of the remaining measurement attributes (structural validity, internal consistency, cultural validity/measurement invariance, stability, measurement error, hypothesis testing validity, and responsiveness)

The remaining psychometric attributes assessed are shown in Table 3. In total, the methodological quality of 192 measurement attributes was rated, 49 measurement properties (25.5%) were very good, 21 (10.9%) were adequate, 35 (18.2%) were doubtful and insufficient methodological quality, and the rest (45.3%) were not assessed.

### Summary of findings

A summary of the survey results is presented in Table 3. NDI, LPDS, FDSD, and ESM-PROM had adequate content validity, but internal consistency is not reported in the NDI's symptom checklist, and FDSD and ESM-PROM did not report structural validity, so only LPDS could receive an A recommendation (adequate content validity and moderate quality evidence of adequate internal consistency). The DHSI and FGI-Checklist are given a Category C recommendation due to high-quality evidence of inadequate psychometric properties. The rest of the scales are recommended for category B. Based on the available evidence, the best PROMs for assessing functional dyspepsia symptoms are the LPDS, a specific scale for the assessment of functional dyspepsia symptoms. It was developed after conducting patient focus group interviews before development.

## Discussion

A total of 24 PROMs from 30 documents were included in this systematic review to assess their psychometric properties according to COSMIN guidelines. LPDS received an A recommendation and can be considered the best tool available for patient self-assessment of functional dyspepsia symptoms. Additional measurement characteristics not currently evaluated should be further investigated in the future to refine the assessment content of the tools.

### Summary of existing literature

FD has been considered a symptom-based disease, and patient self-assessment of its symptoms has important implications for the selection of clinical treatment options. However, there is no consensus on the outcome measures of symptoms. The purpose of this study is not to seek a scale to diagnose or identify FD, but to summarize a self-reporting tool for FD patients' symptoms and to assess symptom changes throughout treatment from a patient's perspective to understand treatment effects. We systematically assessed the psychometric properties of the 24 included PROMs according to the COSMIN guidelines, enriching the current study to some extent. The long recall periods for most tools do not meet the relevant FDA recommendations and are prone to recall bias, which is detrimental to the accuracy of the results. Of the study instruments included in this study, only the modified versions of the SODA, LPDS, FDSD, and PDS symptom scales had a one-day recall period, and the ESM-PROM is a real-time assessment that provides a more realistic response to patients' current symptoms. It is worth noting that the time required to complete most scales is still unclear. In addition, all except the ESM-PROM are assessed on paper, which may be influenced by patient compliance and the prevailing environment.

The NDI is a scale developed by an international group of gastroenterologists and methodologists, which includes components for both symptom assessment and quality of life assessment. Since its development it has been used many times as an outcome indicator in randomized controlled clinical trials and has been widely recognized. The current study concludes that a change in NDI score of at least 10 points may reflect a change in clinical patient symptoms [50]. Some studies have evaluated the psychological measurement characteristics of NDI quality of life subscale, but there is no single evaluation of symptom scale at present [51]. The symptom assessment scale, which provides information on the frequency and severity of patients' symptoms, is an important aspect of assessing the efficacy of treatment regimens and should be further tested for validity and credibility. In addition, the NDI short form SF-NDI was not included in this study because it only evaluates quality of life [52]. Designed and developed for patients with PDS, the LPDS is comprehensive and has good reliability and has been approved by the European Medicines Agency for the assessment of clinical symptoms in patients with PDS. In addition, the entries included in the LPDS also include EPS symptoms such as epigastric pain and epigastric burning, but the validity and reliability of the scale have not been validated in patients with EPS. Further studies should be conducted to understand the variability of the assessment in the two subgroups of the FD population. Both the FDSD and the ESM-PROM were developed under FDA guidance recommendations with very good content validity, and multiple interviews were conducted with patients at the beginning of development to understand patients' perception of symptoms and comprehension of the scale items. They all take less time to fill in the information and use an 11-point numeric rating scale for outcome measures, with the difference that the FDSD has a 1-day recall period but the ESM-PROM is a real-time assessment which is more useful for documenting fluctuations in FD symptoms. Unfortunately, the structural validity and responsiveness of these two instruments have not been reported in studies. Future studies may further investigate in this area.

### Study implications

As a chronic disease, FD is persistent and has a high recurrence rate, which seriously affects the quality of life of patients and imposes an economic burden on patients and their families, as well as putting pressure on the health care system. At present, many scholars have devoted themselves to the research of effective ways to treat FD, but there are great differences in the tools chosen to test the treatment plan, and no consensus has been reached. The selection of sensitive and efficient FD-specific scales is of great importance in the field of clinical gastroenterology, as inappropriate scales are not conducive to detecting the ultimate efficacy of treatment protocols and can even be misleading [53]. To date no studies have used the COSMIN guidelines to systematically evaluate the FD symptom assessment scale. The present study underwent a rigorous screening of the literature to finally summarize 24 specific and non-specific scales that can be used to assess FD symptoms. NDI, PADYQ, LPDS, FDSD, ESM-PROM, a Scale for PDS and SEQ-DYSPEPSIA are all specific scales to assess FD symptoms, and the rest are general gastrointestinal symptom scales. We were guided by the COSMIN guidelines to assess their psychometric properties. According to the available evidence, LPDS is the best tools for self-symptom assessment of FD patients, covering all the core symptoms and additional symptoms of FD and being managed by patients themselves, which is more conducive to reflecting patients' cognition of their own symptoms. Currently unreported measurement attributes can be further explored in future studies to refine the scale assessment content. This study summarizes and evaluates the existing FD efficacy assessment tools from the patient's perspective in order to provide a reference for future clinical studies. Health care providers can choose the most appropriate tool for their study needs.

### Suggestions for future development

#### Real-time symptom assessment

Long instrument recall periods may produce recall bias, and it is recommended to use real-time assessments to understand the dynamics of FD patients' symptoms. The empirical sampling method (ESM), a widely used method in the field of psychiatry, is also important for the real-time symptom assessment of FD patients. An ESM-based method for FD symptom assessment has been reported with significant results. Researchers can install the corresponding program on patients' cell phones, push the content and set reminders at regular intervals every day, and patients only need to complete the assessment content within the reminder time every day [54, 55]. This real-time dynamic assessment helps the investigator to understand the changes in symptoms at each time period and provide personalized treatment. It is less influenced by the environment and location, which results in better patient compliance compared to paper mass scales. In addition, ESM provides insight into the individual characteristics and behavioral patterns of each patient and reduces the cost of investigation. In the future, the relevant analysis of big data can be used to understand the onset of each treatment option and provide personalized treatment in the context of patient differences to help make the right clinical decisions [56]. It is important to note that the number of scales filled out daily should not be too many and the time required to fill them out should not be too long, as this may lead to a decrease in patient compliance and may affect the accuracy of the results. Moreover, investigators can use incentive policies to increase patient response rates and facilitate the smooth conduct of the study. We believe that real-time symptom assessment based on ESM may be an important future development direction, which needs to be thoroughly studied and further explored.

#### Use of pictograms

The validity of pictograms has been recognized in the expert consensus on FD published in Japan and Europe respectively [2, 12]. Some studies have shown that the use of pictograms enhances patients' understanding and memory of verbal representations of the scale, and improves the variability of patient and physician ratings of symptoms [57]. Four of the PROMs included in this study added pictograms with good results: the LPDS and PDS symptom assessment scales both used smiley faces as outcome measures, the FDSD added pictures of the stomach to help patients understand the range of symptoms, and the SEQ-DYSPEPSIA added cartoonish images of symptoms to facilitate patients' understanding of the corresponding symptoms. In addition, the ESM-PROM also suggested that the use of pictograms should be increased in the future. Clinical patients may have some differences in understanding the wording of the scale due to their education and environment. Adding pictures close to life in the scale can help patients better understand the contents of the items and increase the accuracy of the survey results. It should be noted that studies using pictograms are still limited, and even fewer PROMs use them as outcome measures. The validity of pictogram use should be further evaluated in future studies in large sample clinical randomized controlled trials to promote the use of pictograms in FD symptom assessment scales.

### Strengths and limitations of this systematic review

As a non-organic disease, patient self-reporting of symptoms in FD is an important aspect of outcome assessment. However, no studies have systematically evaluated the measurement properties of PROMs in this area based on COSMIN guidelines. The purpose of this study was to systematically evaluate the measurement characteristics of these instruments to recommend the most appropriate tool for assessing symptoms in patients with FD. In this systematic review, we conducted a comprehensive search in four large databases (PubMed, Cochrane, Embase and Web of science), and conducted a search in the list of references included in the study to avoid missing the literature. Then, we use the applied predefined inclusion and exclusion criteria to screen the literature, and used the COSMIN risk of bias checklist, COSMIN quality criteria, COSMIN Modified GRADE ratings to assess the methodological quality, measurement characteristics, and level of evidence of the included studies. Two independent reviewers were evaluated and cross-checked separately, and discrepancies were discussed and agreed upon with a third reviewer. A potential limitation of the systematic review is that although we made detailed screening efforts not to miss any significant studies, omissions may occur. Secondly, the recommendations made by our review do not necessarily imply that PROMs with low recommendation ratings are of unacceptable quality, but only that the symptom content they assessed may be incomplete and require further studies for assessing their measurement characteristics. Finally, we only included PROMs for functional dyspeptic symptoms and did not evaluate other aspects such as quality of life. The clinical can choose the most suitable scale according to the actual needs.

## Conclusion

This systematic review suggests that LPDS is currently the most recommended tool for patient self-assessment of functional dyspepsia symptoms. However, it fails to assess two important areas of cross-cultural validity/ measurement invariance and measurement error. Future studies can build on this foundation and further explore other measurement tools with adequate content validity to find the most appropriate patient self-report tool.

### Supplementary Information

**Additional file 1.**

## Data Availability

All data generated or analysed during this study are included in this published article [and its supplementary information files].
